# Bibliometric Analysis and Research Trend Forecast of Healthy Urban Planning for 40 Years (1981–2020)

**DOI:** 10.3390/ijerph18189444

**Published:** 2021-09-07

**Authors:** Bingyao Jia, Yuting Chen, Jing Wu

**Affiliations:** School of Urban Design, Wuhan University, Wuhan 430072, China; 2017301530047@whu.edu.cn (B.J.); 2018302091004@whu.edu.cn (Y.C.)

**Keywords:** healthy urban planning, spatial planning, bibliometric analysis, CiteSpace, random forest

## Abstract

The history of healthy city planning can be traced back to the beginning of the 19th century. Since the industrialization period, the harsh living conditions of cities and the outbreak of infectious diseases have promoted the coordinated development of urban planning and public health, and people have gradually realized the importance of urban design and planning to the health of residents. After searching keywords related to health city and urban planning, and excluding repeated, non-English, and unrelated papers, this work retrieved 2582 documents as the basic data (timespan is 1 January 1981–31 December 2020, retrieval time is 28 January 2021). Additionally, CiteSpace was used to analyze document co-citation, cooperation network, and topic co-occurrence. Subsequently, random forest algorithm was used to predict the probability of citation. Overall, this work found that the hot spots of healthy urban planning are physical activity, green space, urban green space, and mental health. It also shows the diversification of themes and the development trend of cross-fields in the field of healthy urban planning. In addition, the article found that two factors, namely, the average number of citations of the first author and whether the article belongs to the field of environmental research, have a great impact on the number of citations of the article. This work is of practical significance to relevant practitioners and researchers, because it provides guidance for hot topics and future research directions in the field of healthy urban planning.

## 1. Introduction

The squalid living conditions of industrialized cities and communicable disease outbreaks in the 19th century gave rise to both the urban planning and public health professions [1,2,3], which emphasizes that urban planners, decision makers, and health officials have the responsibility to solve the increasingly serious urban health problems [4,5,6,7,8,9,10,11,12]. Additionally, at the beginning of the 19th century, Canada’s public health committee stated that good urban planning is essential to the preservation of the environment and people’s health [13].

In the 1980s, the World Health Organization (WHO) officially proposed the healthy city project in Europe in 1986 and took five years as a phase to promote the development of healthy cities [14,15]. This project mainly paid attention to health education [16], health equality [17,18], community health [19], health assessment [20], health-related policies [21,22], and project diplomacy [23,24]. Additionally, in terms of urban planning, the initial goals of the project were the introduction of new systems and methods for the construction of healthy cities, and the promotion of various departments fully participating in healthy urban planning.

In the 1990s, with the broad spread of health city projects, more countries formed a cooperative network and integrated healthy city planning into their national policies. Germany further strengthened their health city network at the annual general meeting (AGM) in Greifswald, and emphasized the importance of health impact assessment and urban planning [25]. Korea undertook the enactment of the National Health Promotion Act in 1995, and emphasized in 1998 that at the city level, healthy cities are handled either by the planning department and health department or the planning department and public health center [26].

At the beginning of the 21st century, concepts such as City Health Development Planning (CHDP) [27] and Healthy Urban Planning (HUP) [28], have been proposed successively by the WHO, which emphasized that compared with health service planning, it is more meaningful to deal with urban spatial planning based on the new dynamics of urban management and the principle of the integration of health and urban planning. The concept of healthy urban planning (HUP) was introduced in 2003 by the WHO in The Fourth Phase of the WHO European Healthy Cities Network (2003–2007): Goals and Requirements, which refers to the encouragement and support of city planners to take health into planning strategies and initiative consideration and emphasize equity, well-being, sustainable development, and community safety [15,29]. Additionally, more countries placed urban planning in an important position when formulating their national health policy and laws. Brazil integrated urban planning strategies into The National Health Promotion Policy (PNPS) for healthy city development from 2006 to 2016 [30]. France launched French Act No. 2009-879, French Act No. 2010-788, and French Order No. 2011-210, which promoted its health agency ARS to position itself as a key participant in healthy urban planning [31]. In the past ten years, Sydney, Australia explored systematically how the concept of health as an urban planning issue infiltrated institutional norms of urban strategic planning policy, and realized A Plan for Growing Sydney (APGS) in 2014 [32]. In 2016, China released the Healthy China 2030 plan and emphasized that cities should integrate health into urban planning and design as a first step towards the integration of health into all policies. [33,34].

In terms of research, the themes of healthy city planning are widely distributed, which relates to impact of urban morphology [35,36,37], ecological planning (e.g., urban heat island) [38,39,40,41,42,43,44,45,46,47], and landscape re-source equity [48,49,50,51,52,53,54] on urban residents’ physical and mental health, public health [55,56], and the cross application of urban planning, the perception and evaluation of the urban environment [57,58,59,60,61,62] as well as the space design [63,64] that is based on human behavior. To have a more comprehensive understanding of the research status and development trends in the field of healthy urban planning, grasping the research hotspots and future trends in the numerous literatures is crucial. Hence, the bibliometric and holistic analyses of the macroscopic and the progress of healthy urban planning in the past 40 years are needed. This work will use CiteSpace and the random forest algorithm to show the overall status and future trends of healthy urban planning research from different perspectives, providing researchers with theoretical focus, research frontiers, and a valuable reference in the field of healthy urban planning.

The rest of this paper is organized as follows: In Section 2, we introduce the research design and approach, data collection, and then data analysis. Section 3 illustrates the results in detail, including the CiteSpace part and the random forest part. Section 4 summarizes the whole paper and significant results are discussed in this section. Section 5 concludes this work and plans for future research.

## 2. Data and Methodology

Bibliometric analysis is used for the quantitative analysis of books, articles, or other publications [65,66] and has been applied in various professional fields in recent years to visualize the status, characteristics, evolution, and development trend of knowledge [67,68]. With information development and technology improvement, many visualization tools have emerged in recent years, such as VOSviewer, BibExcel, Sci2, Gephi, and CiteSpace [69,70,71]. These tools can integrate information in the field. However, in the analysis software CiteSpace [72], users can directly use the data downloaded from the Web of Science (WOS) database to set time slices to extract information. Additionally, in comparison with VOSviewer and SCi2, CiteSpace can provide further analyze and complete illustrations, including network betweenness centrality [69]. The co-occurrence network can represent time, frequency, and centrality simultaneously, and the cluster view can use the cluster set extracted from the title, keywords, or abstract [73].

This work will use CiteSpace to conduct a bibliometric analysis on the field of healthy urban planning. To have a deeper understanding of future research trends in this field, this work will also use the random forest algorithm to explore the influencing factors of literature citations using the characteristics of the article (e.g., journal impact factor, author related factors, whether it is a single author, keywords’ density, key field coverage, first author’s country), and key research fields are used to predict hotspots and trends in the field of healthy urban planning.

**Figure 1:**
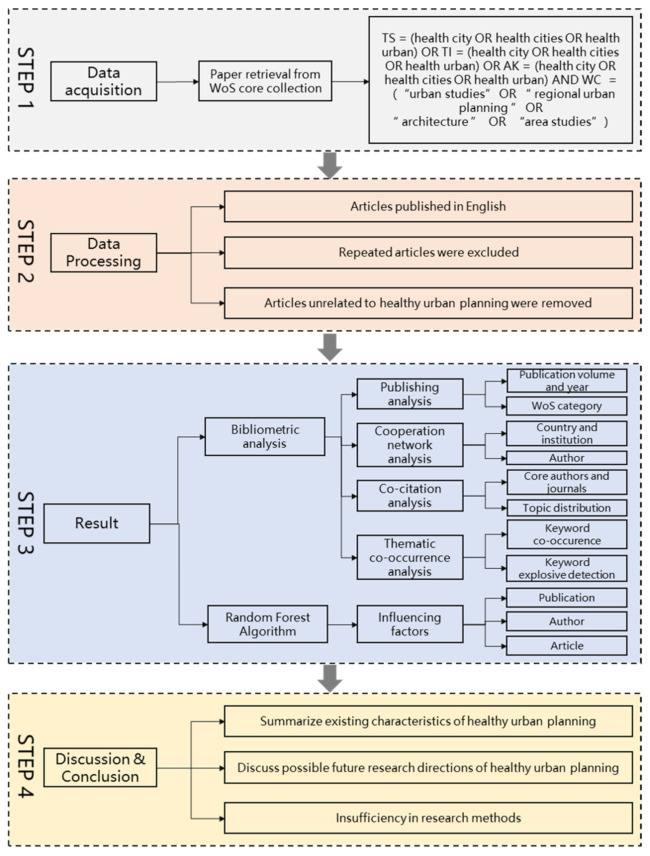
The outline of research design.

In summary, this work combines two different methods to jointly analyze the current and future research focus of healthy urban planning. As for the research design, we presented an outline of the research process in Figure 1, which shows the following four steps of our workflow: data acquisition, data processing, results, and further discussion.

### 2.1. Data Acquisition and Processing

In step one for data acquisition, this work uses the core set of the WOS as the data source. Considering that our research focuses on healthy urban planning, which is the combination of health city and urban planning, we searched for papers of which the research topics, titles, or keywords contain “healthy city” (including synonyms) and the WOS categories that are highly related to urban planning (urban studies, regional urban planning, architecture, and area studies). The specific search formula is TS = (health city OR health cities OR health urban) OR TI = (health city OR health cities OR health urban) OR AK = (health city OR health cities OR health urban) AND WC = (“urban studies” OR “regional urban planning” OR “architecture” OR “area studies”). The time span is 1 January 1981–31 December 2020, and the retrieval time is 28 January 2021.

In step two for data processing, we firstly sorted out the papers of which the document type was articles (including reviews) and excluded non-English papers. Secondly, we removed the repeated articles. Finally, we manually excluded papers that were unrelated to the theme of healthy urban planning; finally, 2582 articles were obtained for CiteSpace analysis. The workflow above followed the criteria of Database Search [74], which is the most used study identification method. Many other bibliometric analyses also follow this strategy [75,76,77]. As for the review articles searched out that related to healthy urban planning, we found that many of them are periodical summaries of the WHO healthy city project [15,28,29,78], some studied city environmental factors and urban health, including factors such as air pollution [79,80], transportation system [81], climate change[82,83], urban green space[84], and indicator system[85,86], and some summarized the research and practice of healthy city planning in different regions or countries such as China[87], America[88], and Brazil[17]. These reviews also help us know more about our research topic.

Then, the random forest algorithm analysis of this work uses the literature data of 2582 articles obtained in the WOS as the basis. The initial data from the WOS for each article included information of the Web of Science Core Collection field tags, such as AU (author), TI (title), etc. We selected terms that could be used in random forest part, reorganized them into 22 forecast influence factors, and divided them into the following three types: publication, author, and document. After calculation and statistics, the influencing factors such as the categories, names, sources, and statistical methods of all the influencing factors, were listed in Table 1. Then, the data were cleaned further. Articles that had anonymous authors (18), or the ones for which the number of citations was 0 (406), along with the ones for which the diversity of research directions was 0 (186), and those whose journal impact factor was 0 (152) were excluded. Finally, 1820 articles were selected for the follow-up predictions.

### 2.2. Research Methods

#### 2.2.1. Bibliometric Method Based on CiteSpace

In this work, we used CiteSpace to conduct bibliometric analysis in the field of healthy urban planning from the aspects of cooperation network at the national, institutional, and individual scales; co-citation analysis of highly cited authors, journals and references; and theme analysis such as keywords co-occurrence.

(1) Analysis process

This research used CiteSpace 5.7.R3 (64-bit)-(c)2003-2021 Chaomei Chen to analyze the co-citation status, cooperation status, and sudden changes (burst detection of words or items through time) of keywords to determine the internal structure of the field, knowledge database of different periods of time, research hotspots, and research frontiers[89,90,91,92,93]. After obtaining the basic information of the literature (e.g., author, title, abstract, keywords, citations, publication journal, organization source, publication year, and publication number) from the WOS database, the data are imported into CiteSpace for deduplication processing and then proceed to the analysis process.

(2) Analysis type

  a. Cooperation Network Analysis

Research on the country and institutional cooperation network is conducive to exploring the spatial and geographic distribution of published articles. Additionally, the author of the study has a key role in reflecting the research ability and evaluating the development of the academic field.

  b. Co-citation Analysis

We must pay attention to the citation frequency of articles to identify the core authors and journals in a certain field. Moreover, from the topic distribution of the reference literature, we can see the knowledge base of the healthy urban planning field and provide reference values for subsequent research.

  c. Thematic Co-occurrence Analysis

The co-occurrence of keywords can reflect the research hotspots of the subject field and provide auxiliary support for scientific research effectively. Additionally, the keyword explosive detection map is to detect keywords with a rapid increase in frequency in the short term, which reflects the occurrence of hot spots more directly.

#### 2.2.2. Random Forest Algorithm

Referring to previous studies [94], we used the random forest algorithm in machine learning to predict articles that may be cited more times in the field of healthy urban planning in the future. This analysis enables this work to determine the factors that have a greater impact on the number of citations in the literature. The appearance of these factors with literature and field characteristics is likely to make the literature more cited in the future. This work will provide healthy urban planning practitioners and scholars with a reference for popular directions in the future.

(1) Modeling process

**Figure 2:**
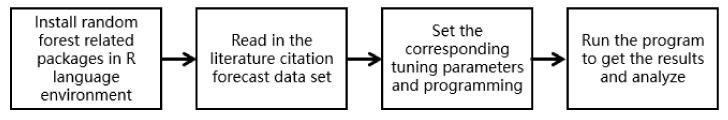
R language modeling process.

Random forest is an ensemble learning algorithm that is based on decision trees, which was proposed in 2001 by Leo Breiman [95] along with Bagging ensemble learning theory [96] with random subspace method [97]. At present, the algorithm is widely used in many fields, such as in biological information [98,99], economics and finance [94,100], computer vision recognition [101], and speech recognition [102]. However, it is still rarely used in the research of urban planning. This work used the R language to implement the random forest prediction model and to comprehensively measure the impact of different influencing factors on the number of citations of the article by calling the random forest algorithm package using the characteristic factors of journals, documents, and authors, such as journal impact factor, author’s writing influence, whether it is a single author, key keyword density, coverage of key areas, the country of the first author, and key research areas. As a supplement to the analysis of CiteSpace, it can predict the research hotspots and trend areas of healthy city planning from a more complete perspective. The specific process is shown in Figure 2.

(2) Algorithm steps
  - Random sampling was performed on the sample data set with replacement to obtain a data set with the same size sample.
  - Specify the mtry value, that is, randomly generate mtry variables for the binary tree on the node, and the choice of the binary tree variables still meets the principle of minimum node impurity.
  - Establish a fully grown decision tree to train all the extracted data sets.
  - The final result is obtained by counting the average of the possible results of all decision trees.

**Figure 3:**
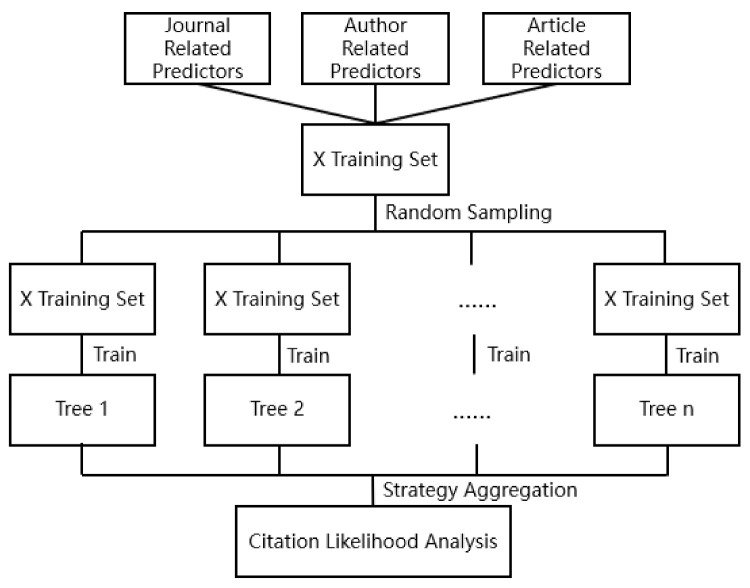
Random forest citation likelihood analysis model.

According to the above steps, we organize the data set and build a random forest model. The model structure is shown in Figure 3. To prevent model overfitting, that is, to reduce the prediction performance of the model in other data sets, this article first randomly divides the literature sample into the following two disjoint sets: the training set (80% of the data) is used to build the model, and the test set (20% of the data) is used to evaluate the performance of the model.

(3) Parameter Selection

  a. Root mean square error (RMSE).

The root mean square error (RMSE) corresponds to the square root of the ratio of the square of the deviation between the predicted value and the true value to the number of observations n. It is used to measure the deviation between the predicted value and the true value. The smaller the value is, the smaller the deviation between the predicted value and the true value is, and the higher the prediction accuracy of the model is. Thus, the prediction model needs to seek to minimize the RMSE of the model training set.

  b. Decision tree (ntree).

This work uses the test set to evaluate the performance of the model for predicting the number of citations in the literature. During the parameter selection process, the number of decision trees (ntree) is fixed to 500 for the following two reasons: first, the more trees exist, the more wasteful the computer’s performance will be. Second, after a certain number of models, the performance of the model remains basically stable, and the improvement of model performance with the increase in the number of trees is very small. Subsequently, this work optimizes the value of the number of predictors (mtry) randomly selected by each tree by searching in discrete intervals of 1…60.

  c. K-fold cross-validation.

To establish a more accurate prediction model under the condition of a smaller amount of data, this paper used K-fold cross-validation to perform k = 10 repeated k-fold cross-validation, which means that the training set is divided into k sub-samples, a single sub-sample is retained as the verification data, the other k-1 samples are used for training, the cross-validation is repeated k times, each sub-sample is verified once, the results are averaged k times, and an optimal result is finally obtained.

**Figure 4:**
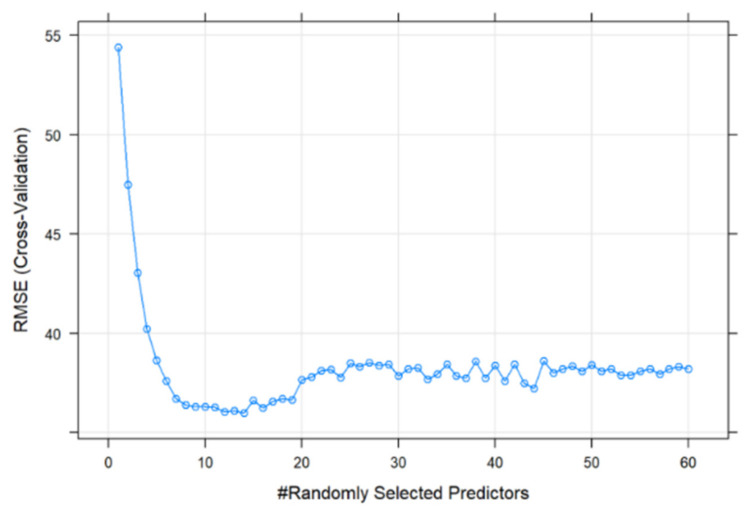
Model selection during training phase, Model selection: in-sample RMSE across different parameters for mtry (number of randomly selected predictors for each tree).

Figure 4 shows the RMSE under different mtry parameter settings. Finally, mtry = 14, which is the parameter selection to minimize RMSE, was selected. On the test set, the optimized model that was run achieved an RMSE of 17.506, which means that the citations predicted by the algorithm may have an error of ±17.506 times, which is only 1.04% relative to the highest citations, thereby proving that the model has an accurate forecast of the number of citations, and the error is within the acceptable range.

  d. Ranking of Influencing Factors

In the random forest prediction model, increased mean squared error (%IncMSE) refers to the expected value of the square of the difference between the estimated value of the parameter and the true value of the parameter caused by the change in the predictive index. The larger the value is, the greater the impact of the factor on the prediction model is. This article uses %IncMSE value (the positive and negative values are only used to represent the degree of influence, and the degree of influence of positive values is greater than the negative value) to determine the factors that have the greatest impact on the number of citations in the literature.

## 3. Result

After data processing, a series of networks were generated to determine the state for healthy urban planning research. The 2582 articles were listed according to their year of publication. Then, networks of country, institution, author, journal, keywords, were derived using CiteSpace. Then, the outcome of random forest algorithm showed the most important influence factor of an article in this field as well as the future trend of the healthy urban planning area.

### 3.1. Publishing Analysis

The analysis of publishing was divided into the following two parts: publication volume and publication year, then the WOS category analysis of the literature. The analysis of the publication volume and the publication year can clearly show the development process of the healthy urban planning area. Additionally, the WOS category analysis of the literature can clearly show the distribution of disciplines in the research field of healthy urban planning.

#### 3.1.1. Publication Volume and Publication Year

**Figure 5:**
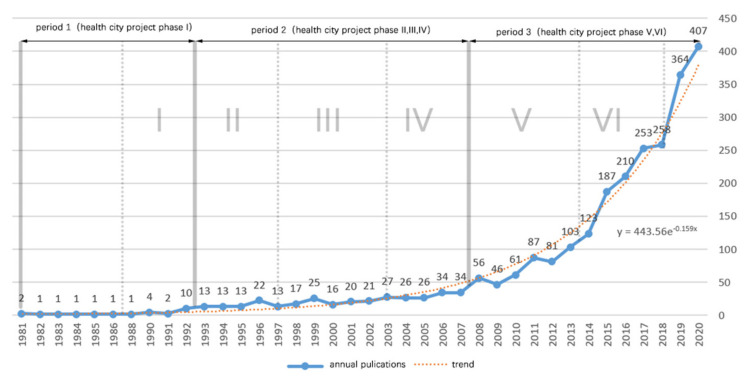
Number of published papers on healthy urban planning (1981–2020).

Figure 5 shows the relationship between the annual publication volume and time of documents related to healthy urban planning for 40 years, that is, from 1981 to 2020, showing an exponential growth as a whole (Trend line function y = 443.56$e^{-0.159x}$). From the figure, the development process of the healthy urban planning can be clearly seen.

According to the growth curve of healthy urban planning research, it is divided into the following three stages: preparation period, budding period, and development period. Notably, its stage node has a corresponding relationship with the WHO healthy city project.

(1) Preparation period 1981–1992 (I). Before 1993, less than 10 articles on healthy urban planning were issued each year, and the growth was extremely slow. This phenomenon may be related to the initial proposal of the concept of a healthy city, and the research was in the exploratory stage.

(2) Budding period 1993–2007 (II, III, IV). A series of research results on healthy urban planning appeared in this period [19,21,103], and some basic theories and empirical research on healthy city planning were presented. At this stage, the number of documents steadily increased, from a minimum of 13 documents per year to a maximum of 34 documents per year in 15 years. A total of 330 articles were published at this stage.

(3) Development period 2008–2020 (V, VI). Since 2008, issues related to healthy cities have gradually become one of the most concerned issues for scientists, government decision makers, various countries, and international organizations in the related fields. Moreover, the number of documents has grown rapidly since 2008, and its growth rate is much greater than the preparation and budding periods. In 2020, 407 related documents were produced, and 2236 documents were produced in 12 years.

In general, the amount of literature related to healthy city planning has increased exponentially in 40 years, and the growth cycle of its literature is basically the same as the development cycle of healthy city projects. The promotion and publicity of healthy city projects have had a certain impact on the academic research of healthy urban planning.

#### 3.1.2. Category Analysis

The exported literature covers 44 WOS categories. Table 2 shows the top 15 subject categories. The distribution of subject categories shows that the field of healthy urban planning places great emphasis on urban, environmental, regional, and geographic issues. In general, healthy city planning research has a strong interdisciplinary nature. In addition to the fields related to urban research, it also has a certain degree of intersection with economics, environmental science, and ecology.

### 3.2. Bibliometric Analysis of CiteSpace Documents on Healthy Urban Planning

There are three analysis types of this work using CiteSpace, including Cooperation network analysis, Co-citation Analysis, and Thematic Co-occurrence Analysis. From these networks, the development context of the healthy urban planning area can be completely presented.

#### 3.2.1. Cooperation Network Analysis

Cooperation networks exist among countries, institutions, as well as authors. We used CiteSpace to build the following three individual networks: a country, an institutional, and an author cooperation network to explore the cooperation relationship within the field of healthy urban planning.

(1) Country Cooperation Network Analysis

**Figure 6:**
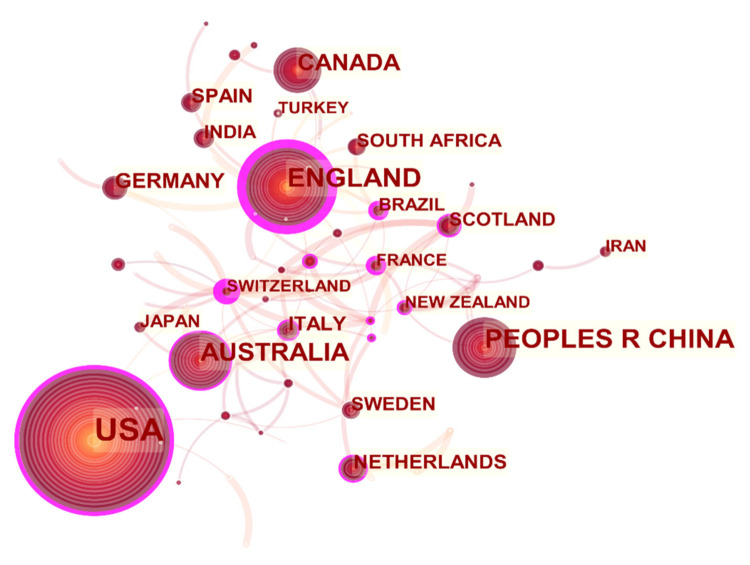
Visualization map of countries participating in healthy urban planning research.

The country cooperation network of healthy urban planning research is shown in Figure 6. The size of the node indicates that the number of articles published in different countries or regions varies. The larger the node is, the more times it appears in the cooperative network. The figure shows that the nodes in the United States, the United Kingdom, China, Australia, and Canada are relatively large, indicating that these countries have made greater contributions to the scientific research cooperation network. In addition to Denmark, Scotland, Finland, Switzerland, and Sweden, China, Brazil, New Zealand, and France are the four countries that have formed a closed and connected graphic, that is, they have formed a certain scale of cooperation. Most other countries have not formed a certain scale of cooperative groups.

Table 3 lists the top 10 countries with the most occurrences in the cooperation network, the time of their first appearance in the cooperation network, as well as their centrality. Notably, the higher the centrality is, the higher the importance of the node is. In terms of centrality: China < Canada, Germany, Spain, and India < Italy < Netherlands < Australia < United States. The centrality of the United Kingdom reaches the maximum of 0.64, which shows that the United Kingdom and the United States, Australia, the Netherlands, and many other countries are in an important position in the cooperation network of healthy city planning.

(2) Institutional Cooperation Network Analysis

**Figure 7:**
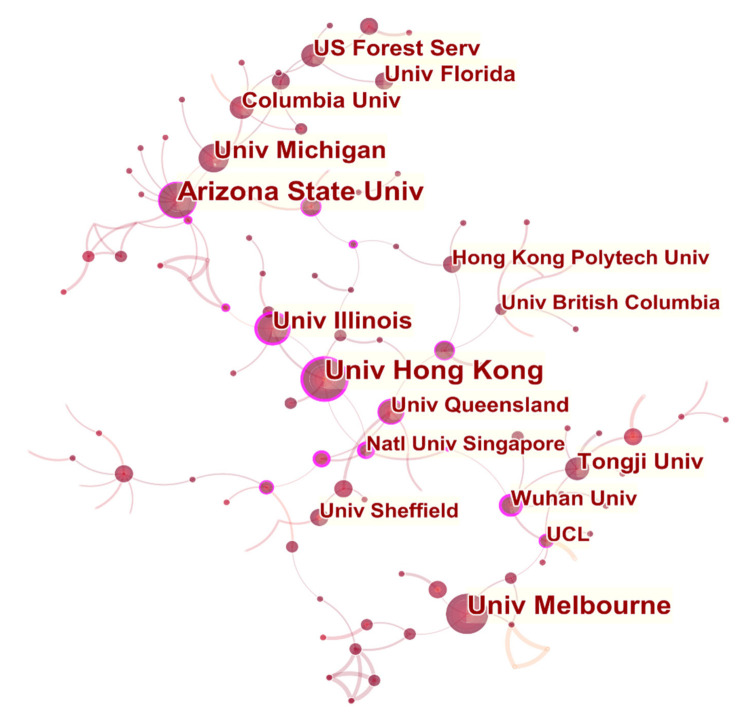
Visualization map of institutions participating in healthy urban planning research.

As shown in Figure 7, some research institutions are relatively concentrated, thereby resulting in some major institution clusters. Few institutions have formed cooperative clusters of a certain scale, but many institutions have strong centrality. The University of Hong Kong (0.3) and University of Illinois (0.24), have high centrality. They are fruitful contributors in this field, with more publications and more in-depth cooperation.

Table 4 lists the top 10 institutions with the most occurrences in the cooperation network. Among the top 10 institutions, seven originated in the United States, and the rest are from China and Australia. This finding reflects the outstanding research results of the United States in the field of healthy urban planning.

(3) Author Cooperation Network Analysis

**Figure 8:**
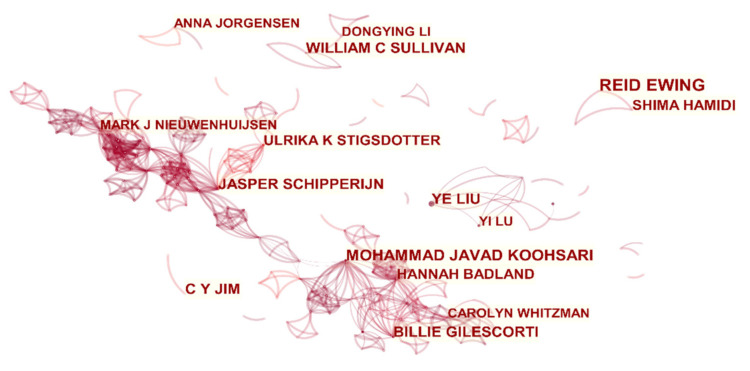
Visualization map of authors participating in healthy urban planning research.

Based on 2582 articles by 6550 different authors, Figure 8 vividly depicts the collaborative network of authors in healthy urban planning. Many authors tend to work with a small group of authors, which leads to several major author groups. For example, the author clusters centered on Ulrika K Stigsdotter, Jasper Schipperijn, Billie Gilescorti, Mohammad Javad Koohsari, etc., all have a closed loop with cross-connections in the figure, which represents the formation of a certain scale of scientific research cooperation. In addition, some authors, such as William C Sullivan and Dongying Li, Yi Liu and Ye Liu, Justin Morgenroth, and Ade Kearns, also have some small-scale scientific research cooperation. Overall, the authors of the healthy urban planning research have formed part of a small-scale collaborative network, but no author has high centrality.

Table 5 lists the top 10 authors and their countries and affiliates with the most appearances in the cooperative network from 1981 to 2020. Among them, Reid Ewing (10) of the University of Utah in the United Kingdom, who first entered the cooperation network in 2008, appeared most frequently in the cooperation network. William C Sullivan (8), C Y Jim (8), Ye Liu (8), Dagmar Haase (7), David J Nowak (7), Ulrika K Stigsdotter (7), Jasper Schipperijn (7), and Billie Gilescorti (7) followed. Most of the authors who are included in the table are in a relatively large-scale cooperation network, and the authors from the United States, China, and Denmark account for the majority from the perspective of country distribution.

#### 3.2.2. Co-Citation Analysis

In this work, we conducted the co-citation analysis from the perspectives of the author, journals, and references, in order to identify the core authors and journals of the healthy urban planning area and the knowledge bases of this area.

(1) Author Co-cited Network Analysis

**Figure 9:**
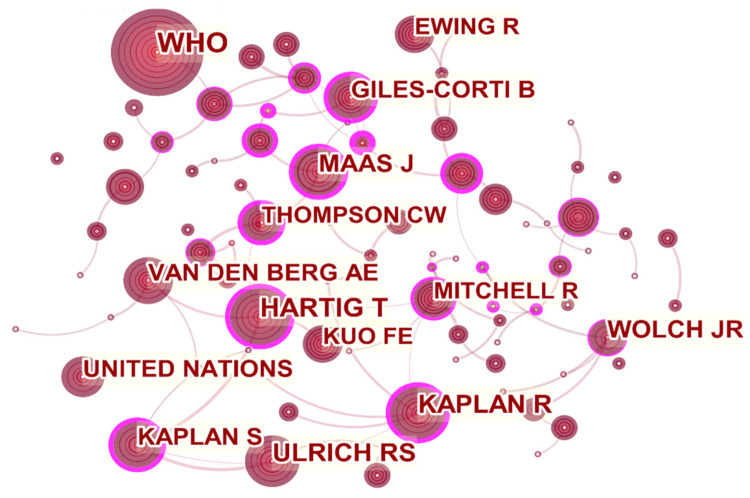
Visualization map of co-cited authors participating in healthy urban planning research.

Figure 9 shows the author co-citation network, where one node represents an author. In the chart generated by CiteSpace, the color corresponds to the year of publication (Figure 9). The lines between authors represent co-citation relationships.

Table 6 lists the top five authors who have been cited. The most cited author is WHO (frequency 237, centrality 0), because WHO, as the proponent of healthy cities and advocates of healthy urban planning, has laid the foundation for the development of the entire discipline. The second place is Harting T (frequency 149, centrality 0.57), the third place is Kaplan R (frequency 131, centrality 0.45), and the fourth place is Ulrich RS (frequency 130, centrality 0.06). They all appeared in “The benefits of nature experience: Improved affect and cognition” [59] as the author of the reference.

(2) Network Analysis of Co-cited Journals

According to the analysis of the co-cited journals, the distribution of the journals cited has a vivid reflection in the field of healthy urban planning.

**Figure 10:**
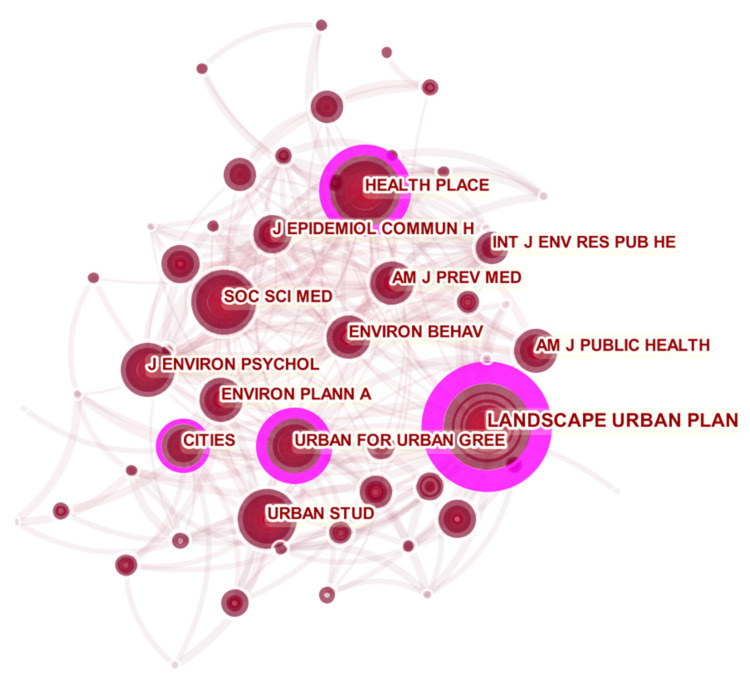
Visualization map of co-citation journal in healthy urban planning research.

What can be seen from Figure 10 and Table 7 is that *Landscape and Urban Planning* is the journal with the most co-citations (812 co-citations, centrality 0.61), and its citations and centrality are much higher than other journals. This result indicates that *Landscape and Urban Planning* has a high reference value for research in the field of healthy urban planning. The second journal is *Health & Place* (529 total citations, centrality 0.32), and the third is *Urban Forestry & Urban Greening* (484 total citations, centrality 0.25). These three journals serve as the core nodes that have established connections with other nodes.

(3) Co-citation Analysis of References

**Figure 11:**
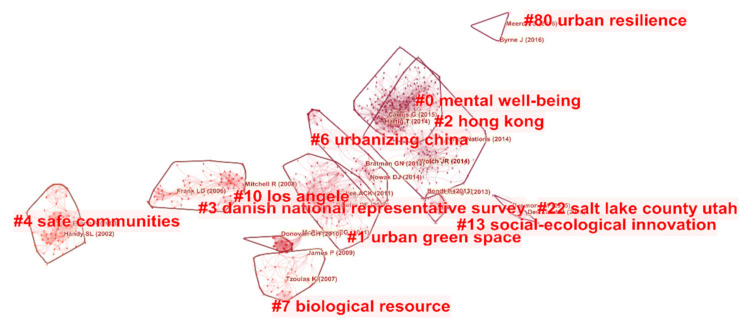
Cluster view of references in healthy urban planning research.

Figure 11 shows a cluster view of references for healthy urban planning and presents the network of co-cited documents. A total of 12 clusters of different sizes are provided in the figure, some of which offer a powerful reference for the study of healthy urban planning. The biggest cluster is #0 mental wellbeing, which focuses on the impact of urban green space landscapes on residents’ psychology [104,105,106,107,108]. The second is #1 urban green spaces, which focuses on the behavior, emotion, health, and spatial quality evaluation of people related to urban green spaces [109,110,111,112,113].The third is #4 safe communities, which focuses on the study of the accessibility characteristics of urban space and its impact on the health of residents [114,115,116,117].

#### 3.2.3. Thematic Co-Occurrence Analysis

Keywords are important in our research of the healthy urban planning area. In this work, we used the co-occurrence of keywords, the timeline of keywords, as well as the burst detection of keywords to identify the hot spots of this research area.

(1) Co-occurrence of Key Words

**Figure 12:**
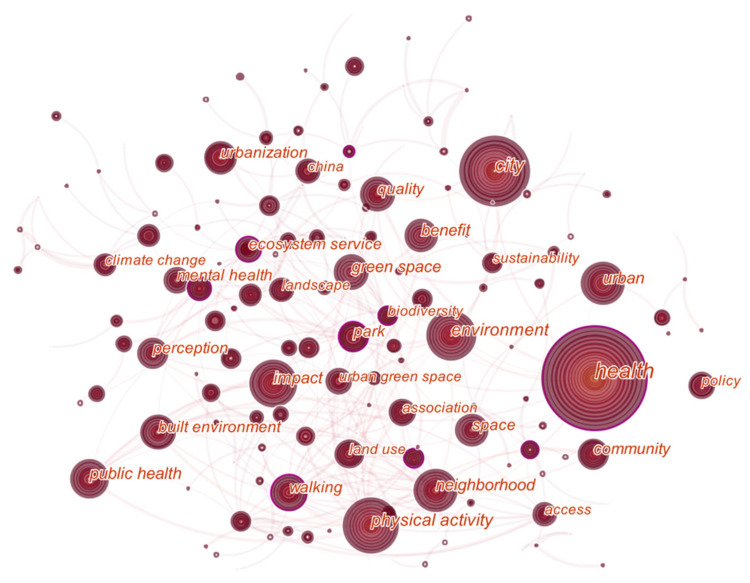
Co-occurrence view of key words in healthy urban planning research.

Figure 12 shows the co-occurrence analysis view of keywords, in which the size of the node represents the frequency of the keyword appearing, and the connection in the node represents the co-occurrence of the keyword in the same document. The more co-occurrences, the thicker the connection, which shows the relevance between keywords. Figure 12 shows the relationship between physical activity and keywords, such as green space, public health, neighborhood, and urban green space, indicating that the research on urban green space is related to human activities, public health, and other topics.

The words health, city, urban, and environment are too broad in meaning to be analyzed. What can be seen from Figure 12 and Table 8 are that the keywords with larger nodes include physical activity (count: 239, centrality: 0.12), neighborhood (count: 147, centrality: 0.08), green space (count: 145, centrality: 0.07), public health (count: 141, centrality: 0.02), and space (count: 120, centrality: 0.09). Centrality means that a node constructs bridges to two unrelated nodes that measures the importance of the nodes in the network. The keywords with greater centrality are more important in the field, but they are not necessarily related to co-occurrence frequency. The keywords with a lower co-occurrence frequency may also have higher centrality. For example, ecosystem service (count: 106, centrality: 0.14) and park (count: 92, centrality: 0.12) also have high centrality. In general, the keyword co-occurrence view provides an objective perspective that shows that the hot spots in the field of healthy urban planning are physical activity, green space, urban green space, and mental health.

(2) Timeline of Key Words

**Figure 13:**
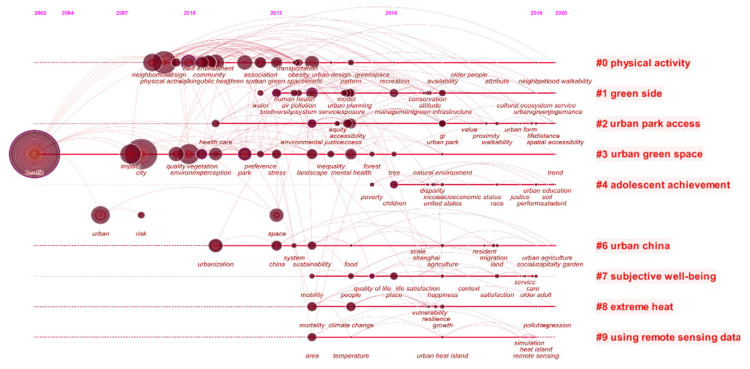
Timeline view of keywords.

Figure 13 depicts a timeline view of keywords that shows the dynamics of keywords in different clusters over time. Overall, the most sustainable cluster is #3 urban green space. From 2002 to 2020, new keywords, including health, city, impact, quality, environment perception, park, preference, stress, landscape, inequality, forest, tree, natural environment, and other key words, have appeared continuously. The research on urban green space also mainly revolves around evaluation, perception, fairness, and other aspects. In addition, #0 physical activity is the largest cluster, and its research content focuses on the community design, accessibility, and residents’ health. The remaining clusters also show other focus points of healthy urban planning, such as accessibility (#2 urban park access), climate issues (#8 extreme heat), group research (#4 adolescent achievement, #7 subjective wellbeing), and new technology (#9 using remote sensing data). In general, the research on healthy urban planning not only focuses on the planning and design of urban space and green space resources but also considers the health factors of urban residents. Moreover, the clustering of keywords has a certain inclusion relationship with the clusters co-cited in the literature, which reflects that the field of healthy urban planning is gradually expanding and becoming diversified.

(3) Burst Detection of Key Words

**Figure 14:**
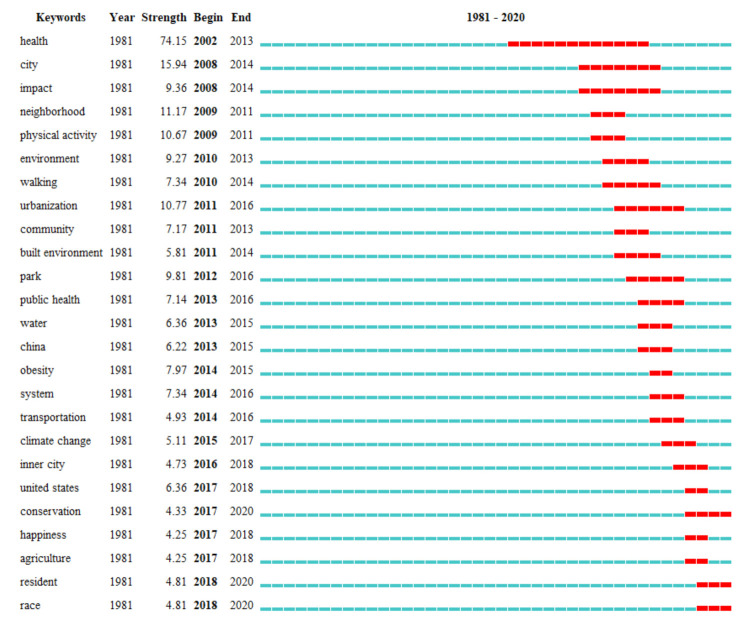
Burst detection of keywords.

Figure 14 illustrates the keyword explosive detection map, which shows the explosive dynamic changes in keywords in the field of healthy urban planning in the past 40 years. In this figure, red color represents the occurrence of burst of keywords, and green color represents no occurrence of burst. In general, the burst of keywords is mainly concentrated in the 20 recent years, which echoes the conclusions in the publication analysis. After removing keywords with broad meanings, such as health and city, the keyword with the longest outbreak time is urbanization (2011–2016). The other keywords have a shorter outbreak time, generally within 3 years, which reflects from the side that the key words in the field of healthy urban planning are changing rapidly and are not sustainable.

### 3.3. Prediction of Citation Possibility of Healthy City Planning Literature

The result of the random forest algorithm of this work can be presented from the following three perspectives that can show the most significant characteristics of this analysis: overall result, the WOS field, and country or region.

#### 3.3.1. Overall Result

Table 9 lists the top 20 influencing factors that have the greatest impact on the number of citations of papers in the field of healthy urban planning. Taken together, the characteristic factors of the document itself have a dominant influence on the number of citations of the document. For example, the %IncMSE value of the number of times the first author’s articles have been cited ranks first, and the total cited impact factor, the number of pages, and whether it is a single author all ranked in the top five. The influence of author and field diversity are also important. The two influencing factors, namely, whether it is a single author and the diversity of paper research direction, also have a higher %IncMSE value, which shows that multi-author cooperation and multi-disciplinary literature have the potential to be highly cited to a certain extent. In addition, the impact of specific areas and national indicators are described below.

#### 3.3.2. WOS Field

Table 10 shows the ranking of influencing factors in the WOS classification. The %IncMSE value of Environmental Studies reached 2.1920207. The existence of this field will greatly increase the frequency of the literature citations. The research on healthy urban planning and the cross-correlation of the environmental field may have a higher citation in the future. The analysis of this article shows that compared with papers related to public management, those related to the environment are the most praised by researchers in terms of the number of citations. In addition, Geography (%IncMSE:0.5209746), Regional and Urban Planning (%IncMSE:0.3672396), and Ecology (%IncMSE:0.1049450) have higher %IncMSE values.

#### 3.3.3. Country or Region

Table 11 shows the ranking of the influencing factors of different countries or regions. What we can derive from the table is that China has the highest %IncMSE value, and other countries have a relatively lower %IncMSE, which may indicate that Chinese researchers have made relatively significant progress in the field of healthy urban planning. However, this does not mean to deny the contribution of any other country in this area. Every country has made great contribution to heathy urban planning research. It is only the objective result produced from the selected 2582 articles.

## 4. Discussion

In terms of the volume of publications, the number of documents related to healthy urban planning has generally shown an exponential increase in 40 years, and the growth cycle of its documents is basically the same as the development cycle of healthy urban projects. The promotion and publicity of healthy city projects has an impact on academic research in healthy urban planning. Then, we found out that the field of healthy urban planning is highly interdisciplinary. In addition to the fields related to urban research, it also has a certain degree of intersection with economics, environmental science, and ecology.

In terms of cooperation networks, at the level of national cooperation, except for Denmark, Scotland, Finland, Switzerland, and Sweden, four other countries, China, Brazil, New Zealand, and France, have each formed a certain scale of cooperation, and most of the other countries have not formed a certain scale of cooperation groups. The United Kingdom, the United States, Australia, the Netherlands, and many other countries play an important role in the cooperation network of healthy urban planning. At the level of institutional cooperation, the University of Hong Kong and University of Illinois are fruitful contributors in this field with a higher number of and more in-depth publications. At the micro level of author cooperation, some small-scale cooperation networks have been formed but without very central authors.

In terms of co-citation analysis, among the authors, WHO, Hartig T, and Kaplan are among the top three in terms of citations. In terms of the journals co-cited, *Landscape and Urban Planning*, *Health & Place*, and *Urban Forestry & Urban Greening* were the core journals in the field of healthy urban planning. In terms of co-cited references, the impact of urban green space landscape on residents’ psychology; the behavior, emotion, health and spatial quality evaluation of people related to urban green space; and the walkability of the space and its impact on the health of residents, partial clustering provides a powerful reference for the study of healthy urban planning.

In terms of thematic co-occurrence, the hot spots in the field of healthy urban planning are physical activity, green space, and mental health. According to the analysis of the keyword timeline, the research of healthy urban planning not only focuses on the planning and design of urban space and green space resources but also considers the health factors of urban residents. Moreover, the clustering of keywords has a certain inclusion relationship with the clusters co-cited in the literature, which reflects that the field of healthy urban planning is gradually expanding and becoming diversified. In addition, through explosive testing, the hot words of healthy urban planning changed rapidly and were not sustainable.

After having a more comprehensive understanding of the field of healthy urban planning, this work used the random forest algorithm to establish a model to predict and rank the factors that influence the citation of literature in the field of healthy urban planning. Taken together, top three influence factors which have dominant influence on the number of citations of the document are: the number of times the first author’s articles have been cited, the number of pages, the journal‘s impact factor. The two influencing factors, namely, whether it is a single author and the diversity of the research direction, also have high %incMSE values, showing that multi-author cooperation and multi-disciplinary literature have the potential of being highly cited to a certain extent. Among the influencing factors related to the WOS fields, the %IncMSE values of Environmental Studies, Geography, Regional and Urban Planning, and Ecology are all high, suggesting that the frequency of the citations of documents which overlap with these fields in healthy urban planning research may increase to some extent. Among the five countries with the largest number of publications in the field of healthy urban planning, China’s %incMSE value ranks first, which may indicate that Chinese researchers have made relatively significant progress in the field of healthy urban planning. Therefore, according to this result, it may be a good choice to cooperate with Chinese researchers. However, this does not mean to deny the contribution of any other country in the research of this field. It is only the objective result produced from the selected 2582 articles. What we really want to do is encourage international cross-country cooperation and enhance the international cooperation network in this field

## 5. Conclusions

Healthy urban planning is a field under rapid development. Over the past few decades, countries have made unremitting efforts to make our cities healthier. In this work, CiteSpace data visualization analysis and a random forest algorithm are used to analyze the articles on the healthy urban planning area in the WOS core database from 1981 to 2020 (as of 31 December 2020). The conclusions are as follows:

(1) The field of healthy city planning has developed rapidly in the past 40 years, the number of annual articles of healthy urban planning research has increased from 2 to 407 exponentially. In the meantime, this field is growing more and more interdisciplinary.

(2) A certain scale of cooperation network has been formed, where the United Kingdom, the United States, Australia, etc., and the University of Hong Kong, University of Illinois, etc., are in an important position. This may be relative to the promotion of the WHO healthy city project. Additionally, *Landscape and Urban Planning*, *Health & Place*, and *Urban Forestry & Urban Greening* are the core journals in the field of healthy urban planning, having the highest co-cited frequency. Moreover, the research hotspots are wide and changing rapidly, mainly focusing on physical activity, urban green space, mental health, etc., which also reflects that the field of healthy city planning is gradually expanding and diversifying.

(3) Based on the analysis result of the random forest algorithm, for related researchers, it is advantageous to consider international cross-country cooperation, interdisciplinary themes, and multi-author cooperation when studying in the field of healthy urban planning in the future, especially considering fields such as Environmental Studies, Geography, Regional and Urban Planning, and Ecology.

The emerging trends and patterns identified using CiteSpace and the prediction results of the random forest algorithm provide novel, interesting, and comprehensive views on how to conduct healthy urban planning research. Based on the conclusion, this article has provided some suggestions for future research: First, the research direction of future healthy urban planning should not only focus on the planning and design of urban space and green space resources but also consider the physical and mental health of urban residents. Specifically, the research on green space needs to consider factors such as human activities and mental health. Second, this study found that the field of healthy urban planning is highly interdisciplinary. At the same time, multi-author cooperation and multi-disciplinary literature have the potential to be highly cited, thereby showing that the field of healthy urban planning is looking forward to the new cross-cutting of knowledge to bring about differences to the field. Healthy urban planning itself involves multi-disciplinary knowledge, such as urban research, geography, environmental science, landscape architecture, public health, and medicine. Strengthening the cross-cooperation among various disciplines and conducting joint research can help analyze the fundamentals of healthy urban development and understand the essential relationship between the city and people’s healthy life and also provide us with multiple perspectives to explore the characteristics of the rules behind the harmonious relationship of people and city.

Despite the contribution of this work, the research still has some shortcomings and limitations. First, the research data are only selected to analyze the literature data in the largest global database of scientific publications (i.e., the WOS), but other international databases [118] are not used. Databases, such as Pubmed and Google Scholar, are not included, which may reduce the comprehensiveness and completeness of the research. Second, when using the random forest model to predict the number of citations, the selection of indicators, such as the number of influencing factors, the number of cross-validation of the training set, and other parameters, can be further optimized through a larger number of experiments to obtain a more accurate model.

## Figures and Tables

**Table 1:** Table of influencing factors.

Category	Name	Field Name in Random Forest Analysis	Name in Metadata from WOS	Sources of Influencing Factors and Statistical Methods
Publication-related factors	Total cited impact factor	Impact_Factor	SO	Data come from the core index of JCR^®^, and the experimental data are the latest publication index data. If the publication is not included in JCR, then its value is set to 0 because it lacks influence.
Author-related factors	The number of times the first author’s articles have been cited	Avg_Ci	AF, Z9	Statistics appear in the data set, the first author of all papers published, and the citation frequency of these papers are added, then it is divided by the total number of papers to determine the number of times the first author’s articles have been cited.
	Nationality of the first author (Select the 5 countries with the largest number of documents)	Name of Countries	C1	Metadata from papers in Web of Science
	Single author	Single_Author	AF	Metadata from papers in Web of Science
Article-related influencing factors	WOS classification of papers (Select the 10 categories with the largest number of documents)	Names of Categories	WC	Metadata from papers in Web of Science
	The number of pages	PG	PG	Metadata from papers in Web of Science
	Number of paper references	NR	NR	Metadata from papers in Web of Science
	The number of times the paper is cited	Z9	Z9	Metadata from papers in Web of Science
	Paper keyword density	KW_dense	DE	Statistics in the data set, the total number of top ten keywords, which are defined as key keywords, the number of key keywords contained in each article is defined as the keyword density of the paper
	Diversity of paper research direction	WC_differ	WC	Statistics of the top 10 WOS categories in the data set are defined as key research directions. The number of key research directions in each article is defined as the diversity of paper research directions.

**Table 2:** Top 15 WOS subject categories based on publications.

Web of Science Category	Rank	Counts
Urban Studies	1	1961
Environmental Studise	2	1289
Regional Urban Planning	3	1061
Geography	4	590
Ecology	5	364
Forestry	6	333
Plant Sciences	7	333
Geography Physical	8	293
Development Studies	9	278
Architecture	10	185
Area Studies	11	164
Economics	12	74
Environmental Sciences	13	72
Biodiversity Conservation	14	70
Green Sustainable Science Technology	15	59

**Table 3:** Top 10 countries based on frequency.

Country	Frequency	Centrality	Year
USA	921	0.24	1992
China	310	0	2003
England	310	0.64	1995
Australia	229	0.61	1995
Canada	147	0.05	1998
Germany	72	0.05	1999
Italy	66	0.11	2003
Netherlands	61	0.14	1995
Spain	58	0.05	2009
India	51	0.05	1999

**Table 4:** Institutions based on publications.

Rank	Institution	Publications	Centrality	Country
1	The University of Hong Kong	41	0.3	China
2	Arizona State University	38	0.17	USA
3	The University of Melbourne	35	0.06	USA
4	The University of Michigan	27	0.08	USA
5	University of Illinois	26	0.24	USA
6	US Forest Service	21	0.05	USA
7	Tongji University	19	0.1	China
8	Columbia University	18	0.06	USA
9	University of Florida	17	0	USA
10	The University of Queensland	16	0.17	Australia

**Table 5:** Top 10 most productive authors in healthy urban planning research: 1981–2020.

Freq	Author	Year	Country	Institution
10	Reid Ewing	2008	UK	University of Utah
9	Mohammad Javad Koohsarik	2009	Iran	University of Tehran
8	William C Sullivan	2009	USA	University of Illinoi
8	C Y Jim	2011	China	University of Hong Kong
7	Ye Liu	2017	China	The Chinese University of Hong Kong
7	Dagmar Haase	2013	Germany	Humboldt Universität zu Berlin
7	David J Nowak	2012	USA	USDA Forest Service
7	Ulrika K Stigsdotter	2010	Denmark	University of Copenhagen
7	Jasper Schipperijn	2010	Denmark	University of Copenhagen
7	Billie Gilescortig	2013	Australia	University of Melbourne

**Table 6:** Top 5 cited authors and their highly cited articles: 1981–2020.

Freq	Centrality	Author	Year	Most Cited Articles	Citations
237	0	WHO	2012	Green justice or just green? Provision of urban green spaces in Berlin, Germany	215
149	0.57	Hartig T	2015	The benefits of nature experience: Improved affect and cognition	218
131	0.45	Kaplan R	2015	The benefits of nature experience: Improved affect and cognition	218
130	0.06	Ulrich RS	2015	The benefits of nature experience: Improved affect and cognition	218
119	0.54	Maas J	2015	Spatial planning for multifunctional green infrastructure: Growing resilience in Detroit	189

**Table 7:** Top 10 productive journals in healthy urban planning research: 1981–2020.

Journal	Frequency	Centrality	Year	Impact Factor
Landscape and Urban Planning	812	0.61	2013	5.441
Health & Place	529	0.32	2014	3.29
Urban Forestry & Urban Greening	484	0.25	2015	4.021
Social Science & Medicine	466	0.06	2014	3.616
Urban Studies	428	0.09	2014	2.828
American Journal of Public Health	362	0.01	2015	6.464
Cities	330	0.17	2016	4.802
Environment and Planning A: Economy and Space	325	0.06	2015	2.855
Journal of Environmental Psychology	322	0.02	2015	2.64
American Journal of Preventive Medicine	306	0.07	2015	4.42

**Table 8:** Top 20 keywords based on count.

Count	Centrality	Year	Keyword	Count	Centrality	Year	Keyword
239	0.12	2009	physical activity	105	0.05	2011	perception
147	0.08	2009	neighborhood	98	0	2015	mental health
145	0.07	2012	green space	96	0.01	2011	urbanization
141	0.02	2011	public health	92	0.12	2012	park
120	0.09	2013	space	90	0.05	2011	land use
118	0	2011	community	88	0.09	2013	urban green space
114	0.02	2011	built environment	87	0.04	2013	china
112	0.04	2010	quality	82	0.03	2014	landscape
110	0.16	2010	walking	78	0.02	2015	climate change
106	0.14	2014	ecosystem service	77	0.05	2015	access

**Table 9:** All the features listed by IncMSE, Feature selection: all the predictors for predicting the number of citations of papers in the healthy urban planning area.

Rank	Field Name	%IncMSE
1	Avg_Ci	42.5297737
2	PG	3.0033313
3	Impact_Factor	3.0017694
4	Environmental_Studies	2.1920207
5	Single_Author	1.9137673
6	WC_differ	1.6044975
7	Peroples_R_China	0.8693223
8	Geography	0.5209746
9	Regional_Urban_Planning	0.3672396
10	Ecology	0.1049450
11	Pbulic_Administration	0.0000000
12	Physical_Geography	0.0000000
13	Development_Studies	−0.1207923
14	NR	−0.3937910
15	Urban_Studies	−0.7585883
16	Plant_Sciences	−1.0216501
17	Forestry	−1.2453836
18	England	−2.3478475
19	USA	−2.3765915
20	Australia	−2.7250626
21	KW_dense	−3.0610388
22	Canada	−3.9821623

**Table 10:** Impactors’ rank (WOS categories).

Rank	WOS Categories	%IncMSE
1	Environmental Studies	2.1920207
2	Geography	0.5209746
3	Regional and Urban Planning	0.3672396
4	Ecology	0.1049450
5	Public Administration	0.0000000
6	Physical Geography	0.0000000
7	Development Studies	−0.1207923
8	Urban Studies	−0.7585883
9	Plant Sciences	−1.0216501
10	Forestry	−1.2453836

**Table 11:** Impactors’ rank (country).

Rank	Country	%IncMSE
1	China	0.8693223
2	England	−2.3478475
3	USA	−2.3765915
4	Australia	−2.7250626
5	Canada	−3.9821623

## Data Availability

Not applicable.

## References

[1] Koohsari M.J., Badland H., Giles-Corti B. (2013). (Re)Designing the built environment to support physical activity: Bringing public health back into urban design and planning. Cities.

[2] Northridge M.E., Sclar E.D., Biswas P. (2003). Sorting out the connections between the built environment and health: A conceptual framework for navigating pathways and planning healthy cities. J. Urban Health-Bull. N. Y. Acad. Med..

[3] Snow J. (1856). On the Mode of Communication of Cholera. Edinb. Med. J..

[4] Wang H.J., He Q.Q., Liu X.J., Zhuang Y.H., Hong S. (2012). Global urbanization research from 1991 to 2009: A systematic research review. Landsc. Urban Plan..

[5] Tan Y., Xu H., Zhang X. (2016). Sustainable urbanization in China: A comprehensive literature review. Cities.

[6] Moore M., Gould P., Keary B.S. (2003). Global urbanization and impact on health. Int. J. Hyg. Environ. Health.

[7] Mutatkar R.K. (1995). Public-health problems of urbanization. Soc. Sci. Med..

[8] Gong P., Liang S., Carlton E.J., Jiang Q., Wu J., Wang L., Remais J.V. (2012). Urbanisation and health in China. Lancet.

[9] Cyril S., Oldroyd J.C., Renzaho A. (2002). Urbanisation, urbanicity, and health: A systematic review of the reliability and validity of urbanicity scales. BMC Public Health.

[10] Connolly C., Keil R., Ali S.H. (2021). Extended urbanisation and the spatialities of infectious disease: Demographic change, infrastructure and governance. Urban Stud..

[11] Alirol E., Getaz L., Stoll B., Chappuis F., Loutan L. (2011). Urbanisation and infectious diseases in a globalised world. Lancet Infect. Dis..

[12] Swann W.L., Brixey E., Wohler W. (2021). Linking local sustainability policies to health outcomes: An analysis of the urban sustainability-health nexus. J. Urban Aff..

[13] Macfarlane R.G., Wood L.P., Campbell M.E. (2015). Healthy Toronto by Design: Promoting a healthier built environment. Can. J. Public Health-Rev. Can. De Sante Publique.

[14] Ashton J., Grey P., Barnard K. (1986). Healthy cities—WHO’s New Public Health initiative. Health Promot..

[15] Tsouros A.D. (2015). Twenty-seven years of the WHO European Healthy Cities movement: A sustainable movement for change and innovation at the local level. Health Promot. Int..

[16] Ashton J. (1991). The healthy cities project—A challenge for health-education. Health Educ. Q..

[17] Stephens C., Akerman M., Avle S., Maia P.B., Campanario P., Doe B., Tetteh D. (1997). Urban equity and urban health: Using existing data to understand inequalities in health and environment in Accra, Ghana and Sao Paulo, Brazil. Environ. Urban..

[18] Ritsatakis A., Ostergren P.O., Webster P. (2015). Tackling the social determinants of inequalities in health during Phase V of the Healthy Cities Project in Europe. Health Promot. Int..

[19] Flynn B.C., Ray D.W., Rider M.S. (1994). Empowering Communities—Action Research through Healthy Cities. Health Educ. Q..

[20] Webster P., Lipp A. (2009). The evolution of the WHO city health profiles: A content review. Health Promot. Int..

[21] Werna E., Harpham T., Blue I., Goldstein G. (1999). From healthy city projects to healthy cities. Environ. Urban..

[22] Montiel R.P., Barten F. (1999). Urban governance and health development in Leon, Nicaragua. Environ. Urban..

[23] Green G., Price C., Lipp A., Priestley R. (2009). Partnership structures in the WHO European Healthy Cities project. Health Promot. Int..

[24] de Leeuw E. (2017). Healthy Cities are back! (They were never gone). Health Promot. Int..

[25] Plumer K.D., Kennedy L., Trojan A. (2010). Evaluating the implementation of the WHO Healthy Cities Programme across Germany (1999–2002). Health Promot. Int..

[26] Moon J.Y., Nam E.W., Dhakal S. (2014). Empowerment for Healthy Cities and Communities in Korea. J. Urban Health-Bull. N. Y. Acad. Med..

[27] Green G., Acres J., Price C., Tsouros A. (2009). City health development planning. Health Promot. Int..

[28] Barton H., Grant M. (2011). Urban Planning for Healthy Cities A Review of the Progress of the European Healthy Cities Programme. J. Urban Health-Bull. N. Y. Acad. Med..

[29] WHO Regional Office for Europe (2003). Phase IV (2003–2008) WHO European Healthy Cities Network: Goals and Requirements.

[30] Girotti Sperandio A.M., Francisco Filho L.L., Mattos T.P. (2016). Health promotion policy and urban planning: Joint efforts for the development of healthy cities. Cienc. Saude Coletiva.

[31] Le Gall A.R., Lemaire N., Jabot F. (2018). Lessons learned from co-constructing a guide on healthy urban planning and on integrating health issues into environmental impact assessments conducted on French urban development projects. Impact Assess. Proj. Apprais..

[32] Harris P., Kent J., Sainsbury P., Riley E., Sharma N., Harris E. (2020). Healthy urban planning: An institutional policy analysis of strategic planning in Sydney, Australia. Health Promot. Int..

[33] Yang J., Siri J.G., Remais J.V., Cheng Q., Zhang H., Chan K.K.Y., Sun Z., Zhao Y., Cong N., Li X. (2018). The Tsinghua-Lancet Commission on Healthy Cities in China: Unlocking the power of cities for a healthy China. Lancet.

[34] Summerskill W., Wang H.H., Horton R. (2018). Healthy cities: Key to a healthy future in China. Lancet.

[35] Hamidi S., Ewing R. (2014). A longitudinal study of changes in urban sprawl between 2000 and 2010 in the United States. Landsc. Urban Plan..

[36] Dovey K., Pafka E. (2019). What is walkability? The urban DMA. Urban Stud..

[37] Debbage N., Shepherd J.M. (2015). The urban heat island effect and city contiguity. Comput. Environ. Urban Syst..

[38] Wu J.G., Xiang W.N., Zhao J.Z. (2014). Urban ecology in China: Historical developments and future directions. Landsc. Urban Plan..

[39] Chun B., Guldmann J.M. (2018). Impact of greening on the urban heat island: Seasonal variations and mitigation strategies. Comput. Environ. Urban Syst..

[40] Zhang Y.J., Murray A.T., Turner B.L. (2017). Optimizing green space locations to reduce daytime and nighttime urban heat island effects in Phoenix, Arizona. Landsc. Urban Plan..

[41] Kabisch N., Haase D. (2013). Green spaces of European cities revisited for 1990-2006. Landsc. Urban Plan..

[42] Selmi W., Weber C., Riviere E., Blond N., Mehdi L., Nowak D. (2016). Air pollution removal by trees in public green spaces in Strasbourg city, France. Urban For. Urban Green..

[43] McPherson E.G., Simpson J.R., Xiao Q.F., Wu C.X. (2011). Million trees Los Angeles canopy cover and benefit assessment. Landsc. Urban Plan..

[44] Meerow S., Newell J.P. (2017). Spatial planning for multifunctional green infrastructure: Growing resilience in Detroit. Landsc. Urban Plan..

[45] Douglas O., Lennon M., Scott M. (2017). Green space benefits for health and well-being: A life-course approach for urban planning, design and management. Cities.

[46] MacLachlan A., Biggs E., Roberts G., Boruff B. (2021). Sustainable City Planning: A Data-Driven Approach for Mitigating Urban Heat. Front. Built Environ..

[47] Ding Y., Zhang L. Research on Healthy Urban Design Using Remote Sensing Technique. Proceedings of the 6th International Symposium on Project Management.

[48] Xiao Y., Wang Z., Li Z.G., Tang Z.L. (2017). An assessment of urban park access in Shanghai—Implications for the social equity in urban China. Landsc. Urban Plan..

[49] Anguelovski I., Connolly J.J.T., Masip L., Pearsall H. (2017). Assessing green gentrification in historically disenfranchised neighborhoods: A longitudinal and spatial analysis of Barcelona. Urban Geogr..

[50] Wustemann H., Kalisch D., Kolbe J. (2017). Access to urban green space and environmental inequalities in Germany. Landsc. Urban Plan..

[51] Rigolon A., Browning M., Jennings V. (2018). Inequities in the quality of urban park systems: An environmental justice investigation of cities in the United States. Landsc. Urban Plan..

[52] Dai D. (2011). Racial/ethnic and socioeconomic disparities in urban green space accessibility: Where to intervene?. Landsc. Urban Plan..

[53] Wolch J.R., Byrne J., Newell J.P. (2014). Urban green space, public health, and environmental justice: The challenge of making cities ‘just green enough’. Landsc. Urban Plan..

[54] Xing L.J., Liu Y.F., Wang B.S., Wang Y.H., Liu H.J. (2020). An environmental justice study on spatial access to parks for youth by using an improved 2SFCA method in Wuhan, China. Cities.

[55] Rice L. (2020). After Covid-19: Urban design as spatial medicine. Urban Des. Int..

[56] Hamidi S., Sabouri S., Ewing R. (2020). Does Density Aggravate the COVID-19 Pandemic? Early Findings and Lessons for Planners. J. Am. Plan. Assoc..

[57] Grahn P., Stigsdotter U.K. (2010). The relation between perceived sensory dimensions of urban green space and stress restoration. Landsc. Urban Plan..

[58] Van den Berg A.E., Jorgensen A., Wilson E.R. (2014). Evaluating restoration in urban green spaces: Does setting type make a difference?. Landsc. Urban Plan..

[59] Bratman G.N., Daily G.C., Levy B.J., Gross J.J. (2015). The benefits of nature experience: Improved affect and cognition. Landsc. Urban Plan..

[60] Klemm W., Heusinkveld B.G., Lenzholzer S., van Hove B. (2015). Street greenery and its physical and psychological impact on thermal comfort. Landsc. Urban Plan..

[61] Thompson C.W., Roe J., Aspinall P., Mitchell R., Clow A., Miller D. (2012). More green space is linked to less stress in deprived communities: Evidence from salivary cortisol patterns. Landsc. Urban Plan..

[62] Huang Q.Y., Yang M.Y., Jane H.A., Li S.H., Bauer N. (2019). Trees, grass, or concrete? The effects of different types of environments on stress reduction. Landsc. Urban Plan..

[63] Brown G., Schebella M.F., Weber D. (2014). Using participatory GIS to measure physical activity and urban park benefits. Landsc. Urban Plan..

[64] Kerr J., Rosenberg D., Frank L. (2012). The Role of the Built Environment in Healthy Aging: Community Design, Physical Activity, and Health among Older Adults. J. Plan. Lit..

[65] Ellegaard O., Wallin J.A. (2015). The bibliometric analysis of scholarly production: How great is the impact?. Scientometrics.

[66] Price D.J.D. (1976). General theory of bibliometric and other cumulative advantage processes. J. Am. Soc. Inf. Sci..

[67] Bornmann L., Mutz R. (2015). Growth rates of modern science: A bibliometric analysis based on the number of publications and cited references. J. Assoc. Inf. Sci. Technol..

[68] Mingers J., Leydesdorff L. (2015). A review of theory and practice in scientometrics. Eur. J. Oper. Res..

[69] Yang J., Cheng C., Shen S., Yang S. Comparison of Complex Network Analysis Software: Citespace, SCI2 and Gephi. Proceedings of the 2017 IEEE 2nd International Conference on Big Data Analysis (ICBDA).

[70] Ruas T.L., Pereira L. (2014). Como construir indicadores de Ciência, Tecnologia e Inovação usando Web of Science, Derwent World Patent Index, Bibexcel e Pajek?. Perspect. Em Ciência Da Inf..

[71] van Eck N.J., Waltman L. VOSviewer: A Computer Program for Bibliometric Mapping. Proceedings of Issi 2009—12th International Conference of the International Society for Scientometrics and Informetrics.

[72] Chen C.M. (2006). CiteSpace II: Detecting and visualizing emerging trends and transient patterns in scientific literature. J. Am. Soc. Inf. Sci. Technol..

[73] Niazi M.A. (2016). CiteSpace: A Practical Guide for Mapping Scientific Literature. Complex Adapt. Syst. Modeling.

[74] Harari M.B., Parola H.R., Hartwell C.J., Riegelman A. (2020). Literature searches in systematic reviews and meta-analyses: A review, evaluation, and recommendations. J. Vocat. Behav..

[75] He K., Zhang J.B., Zeng Y.M. (2019). Knowledge domain and emerging trends of agricultural waste management in the field of social science: A scientometric review. Sci. Total Environ..

[76] Zhu J., Song L.J., Zhu L., Johnson R.E. (2019). Visualizing the landscape and evolution of leadership research. Leadersh. Q..

[77] Zhong B., Wu H., Ding L., Love P.E.D., Li H., Luo H., Jiao L. (2019). Mapping computer vision research in construction: Developments, knowledge gaps and implications for research. Autom. Constr..

[78] WHO Healthy Cities Project Office (2009). Goals and Requirements—Phase V: (2009–2013). https://www.euro.who.int/__data/assets/pdf_file/0009/100989/E92260.pdf.

[79] Nieuwenhuijsen M.J. (2020). Urban and transport planning pathways to carbon neutral, liveable and healthy cities; A review of the current evidence. Environ. Int..

[80] Khomenko S., Nieuwenhuijsen M., Ambros A., Wegener S., Mueller N. (2020). Is a liveable city a healthy city? Health impacts of urban and transport planning in Vienna, Austria. Environ. Res..

[81] Louro A., da Costa N.M., da Costa E.M. (2019). Sustainable Urban Mobility Policies as a Path to Healthy CitiesThe Case Study of LMA, Portugal. Sustainability.

[82] Bentley M. (2007). Healthy Cities, local environmental action and climate change. Health Promot. Int..

[83] Gao S., Zhang H.Q. (2020). Urban planning for low-carbon sustainable development. Sust. Comput..

[84] Nieuwenhuijsen M.J., Khreis H., Triguero-Mas M., Gascon M., Dadvand P. (2017). Fifty Shades of Green Pathway to Healthy Urban Living. Epidemiology.

[85] Capolongo S., Buffoli M., Brambilla A., Rebecchi A. (2020). Healthy urban planning and design strategies to improve urban quality and attractiveness of places. TECHNE.

[86] Pineo H., Glonti K., Rutter H., Zimmermann N., Wilkinson P., Davies M. (2020). Use of Urban Health Indicator Tools by Built Environment Policy- and Decision-Makers: A Systematic Review and Narrative Synthesis. J. Urban Health-Bull. N. Y. Acad. Med..

[87] Wang L., JIANG X.J., Ye D. (2020). Research Hotspots and Progress on Healthy City Planning in China: A Bibliometric Analysis Based on Citespace. Urban Dev. Stud..

[88] Freudenberg N. (2000). Health promotion in the city: A review of current practice and future prospects in the United States. Annu. Rev. Public Health.

[89] Chen C.M., Hu Z.G., Liu S.B., Tseng H. (2012). Emerging trends in regenerative medicine: A scientometric analysis in CiteSpace. Expert Opin. Biol. Ther..

[90] Wei F.W., Grubesic T.H., Bishop B.W. (2015). Exploring the GIS Knowledge Domain Using CiteSpace. Prof. Geogr..

[91] Li X.J., Ma E., Qu H.L. (2017). Knowledge mapping of hospitality research—A visual analysis using CiteSpace. Int. J. Hosp. Manag..

[92] Liang Y.D., Li Y., Zhao J., Wang X.Y., Zhu H.Z., Chen X.H. (2017). Study of acupuncture for low back pain in recent 20 years: A bibliometric analysis via CiteSpace. J. Pain Res..

[93] Fang Y., Yin J., Wu B.H. (2018). Climate change and tourism: A scientometric analysis using CiteSpace. J. Sustain. Tour..

[94] Tabak B.M., Silva T.C., Fiche M.E., Braz T. (2021). Citation likelihood analysis of the interbank financial networks literature: A machine learning and bibliometric approach. Phys. A.

[95] Breiman L. (2001). Random forests. Mach. Learn..

[96] Kwok S.W., Carter C. (2013). Multiple decision trees. arXiv.

[97] Ho T.K. (1998). The random subspace method for constructing decision forests. IEEE Trans. Pattern Anal. Mach. Intell..

[98] Acharjee A., Kloosterman B., Visser R.G.F., Maliepaard C. (2016). Integration of multi-omics data for prediction of phenotypic traits using random forest. BMC Bioinform..

[99] Svetnik V., Liaw A., Tong C., Culberson J.C., Sheridan R.P., Feuston B.P. (2003). Random forest: A classification and regression tool for compound classification and QSAR modeling. J. Chem. Inf. Comput. Sci..

[100] Cutler D.R., Edwards T.C., Beard K.H., Cutler A., Hess K.T. (2007). Random forests for classification in ecology. Ecology.

[101] Lindner C., Bromiley P.A., Ionita M.C., Cootes T.F. (2015). Robust and Accurate Shape Model Matching Using Random Forest Regression-Voting. IEEE Trans. Pattern Anal. Mach. Intell..

[102] Baumann T. Decision Tree Usage For Incremental Parametric Speech Synthesis. Proceedings of the 2014 IEEE International Conference on Acoustics, Speech and Signal Processing (ICASSP).

[103] Webster P. (1999). Review of the “City Health Profiles” produced by WHO Healthy Cities—do they present information on health and its determinants and what are their perceived benefits?. J. Epidemiol. Community Health.

[104] Akpinar A. (2016). How is quality of urban green spaces associated with physical activity and health?. Urban For. Urban Green..

[105] Hodson C.B., Sander H.A. (2017). Green urban landscapes and school-level academic performance. Landsc. Urban Plan..

[106] Ferguson M., Roberts H.E., McEachan R.R.C., Dallimer M. (2018). Contrasting distributions of urban green infrastructure across social and ethno-racial groups. Landsc. Urban Plan..

[107] Vujcic M., Tomicevic-Dubljevic J., Zivojinovic I., Toskovic O. (2019). Connection between urban green areas and visitors’ physical and mental well-being. Urban For. Urban Green..

[108] Cameron R.W.F., Brindley P., Mears M., McEwan K., Ferguson F., Sheffield D., Jorgensen A., Riley J., Goodrick J., Ballard L. (2020). Where the wild things are! Do urban green spaces with greater avian biodiversity promote more positive emotions in humans?. Urban Ecosyst..

[109] Gidlow C.J., Ellis N.J., Bostock S. (2012). Development of the Neighbourhood Green Space Tool (NGST). Landsc. Urban Plan..

[110] Zhang H., Chen B., Sun Z., Bao Z. (2013). Landscape perception and recreation needs in urban green space in Fuyang, Hangzhou, China. Urban For. Urban Green..

[111] Adinolfi C., Patricia Suarez-Caceres G., Carinanos P. (2014). Relation between visitors’ behaviour and characteristics of green spaces in the city of Granada, south-eastern Spain. Urban For. Urban Green..

[112] Hunter A.J., Luck G.W. (2015). Defining and measuring the social-ecological quality of urban greenspace: A semi-systematic review. Urban Ecosyst..

[113] Eisenman T.S. (2016). Greening Cities In An Urbanizing Age The Human Health Bases in the Nineteenth and Early Twenty-first Centuries. Chang. Over Time-Int. J. Conserv. Built Environ..

[114] Doyle S., Kelly-Schwartz A., Schlossberg M., Stockard J. (2006). Active community environments and health—The relationship of walkable and safe communities to individual health. J. Am. Plan. Assoc..

[115] Frank L.D., Sallis J.F., Conway T.L., Chapman J.E., Saelens B.E., Bachman W. (2006). Many pathways from land use to health—Associations between neighborhood walkability and active transportation, body mass index, and air quality. J. Am. Plan. Assoc..

[116] Vojnovic I., Jackson-Elmoore C., Holtrop J., Bruch S. (2006). The renewed interest in urban form and public health: Promoting increased physical activity in Michigan. Cities.

[117] Pretty J., Peacock J., Hine R., Sellens M., South N., Griffin M. (2007). Green exercise in the UK countryside: Effects on health and psychological well-being, and implications for policy and planning. J. Environ. Plan. Manag..

[118] Yang L., Chen Z., Liu T., Gong Z., Yu Y., Wang J. (2013). Global trends of solid waste research from 1997 to 2011 by using bibliometric analysis. Scientometrics.

